# Spectral analysis of ECG and SpO₂ for machine learning classification of Sleep-Disordered breathing

**DOI:** 10.1007/s11325-026-03602-5

**Published:** 2026-02-11

**Authors:** Zachary B. Strumpf, Farhad Kaffashi, Susheel P. Patil, Kingman P. Strohl, Kenneth A. Loparo, Frank J. Jacono

**Affiliations:** 1 Division of Pulmonary, Critical Care and Sleep Medicine, University Hospitals Cleveland Medical Center, 11100 Euclid Avenue, Bolwell, Cleveland, OH 44106 USA; 2 School of Medicine, Case Western Reserve University, Cleveland, USA; 3 ISSACS: Institute for Smart, Secure and Connected Systems, Case Western Reserve University, Cleveland, USA

**Keywords:** Sleep-disordered breathing, Spectral analysis, Machine learning, Inpatient diagnosis

## Abstract

**Purpose:**

Sleep-disordered breathing (SDB), particularly obstructive sleep apnea (OSA), is prevalent in hospitalized patients, yet remains underdiagnosed due to limited screening tools. This pilot study aimed to develop and validate a machine-learning classifier using electrocardiogram (ECG) and pulse oximetry (SpO₂) waveforms to detect SDB, leveraging routinely collected physiological data to enable non-invasive, scalable screening in hospitalized patients.

**Methods:**

We utilized data from the Multi-Ethnic Study of Atherosclerosis (MESA) Sleep cohort, which included full overnight polysomnography (PSG). A total of 122 participants were randomly selected, enriched for severe OSA cases. Spectral analysis of R-R intervals and SpO₂ variability was performed, and a support vector machine (SVM) classifier was trained using a subset of subjects (*n* = 30). Performance was evaluated in a validation set (*n* = 92) using standard classification metrics with 95% CIs calculated.

**Results:**

The first-stage classifier (SVM-1) demonstrated high sensitivity (98.7%) and specificity (99.0%) in identifying respiratory events at the window level. The second-stage classifier (SVM-2) correctly classified OSA presence with 100% accuracy in the training set. When applied to the validation set, the combined model achieved a sensitivity of 72.3%, specificity of 73.3%, and F1-score of 73.1%. Performance was impacted by arrhythmias, and lack of direct respiratory effort measures.

**Conclusion:**

A machine-learning model using routinely collected ECG and SpO₂ data shows promise for SDB detection but may require additional cardiopulmonary parameters for clinical decision-making in the ICU. Further validation with data collected in real-world ICU settings is warranted.

## Introduction

Sleep-related breathing disorders (SDB), particularly obstructive sleep apnea (OSA), are common, treatable conditions with significant health implications. Moderate-to-severe OSA, affecting an estimated 24 million individuals in the U.S., is closely associated with adverse health outcomes, including cardiovascular disease, stroke, and increased all-cause mortality [1–4]. Furthermore, severity of OSA correlates with hypoxemia and hypoventilation, both of which are linked to poor outcomes [5]. Despite the availability of validated outpatient screening tools, most cases remain undiagnosed due to barriers in case management, limited use of screening in practice, and low public awareness [6, 7]. Recent publications underscore the critical need for expanded diagnostic settings and innovative methods to improve identification and management of patients in whom treatment is indicated [8, 9].

Hospitalizations, particularly to the Intensive Care Unit (ICU), are clinical settings with a high prevalence of SDB [10–12]. Severe forms of SDB in the ICU are independently associated with adverse outcomes during hospitalization and after discharge [13–16], yet many affected patients remain undiagnosed and unmanaged at discharge. Studies of ICU populations have applied both gold-standard and novel signal processing and analytic methods for SDB diagnosis [17, 18]. However, many of these outpatient-oriented tools rely not only on physician awareness but also on cumbersome equipment, like polysomnography, or proprietary research platforms, limiting widespread applicability. In-hospital diagnostics are equally challenging due to limited measurements of key SDB physiological variables, limited technical and decision-making tools, and the dynamic nature of critical care settings. However, appropriate and timely identification of at-risk patients has the potential to reduce healthcare costs and improve patient outcomes by facilitating enrollment of patients in coordinated care pathways.

Physiological signal analysis of routinely collected ICU data could be feasible to facilitate recognition and management of patients with OSA. Novel analytic platforms, primarily tested in research or outpatient settings, have shown accuracy in identifying OSA using either single- or multiple-channel analytic methodologies [19–21]. ICU patients typically undergo continuous monitoring of heart rate, oxygen saturation, and respiratory rate, presenting an opportunity for diagnostic methods utilizing patient data that is routinely collected and can be archived for post processing. A small ICU study demonstrated the feasibility of SDB detection but highlighted the need for respiratory effort measures [18, 21]. Addressing these issues would aid in identification of SDB in ICU patients by leveraging existing data without additional equipment or staffing burdens.

The purpose of the study was to simulate two-channel ICU monitoring and utilize data from large, population-based sleep research cohorts to apply machine-learning-based clinical decision support (CDS) tools that could be applied to OSA risk identification decision-making. In particular, we developed and optimized an analytical platform based on processing of the electrocardiogram (ECG) and pulse oximetry (SpO2) waveforms to drive probability estimates useful in OSA identification. Prior ECG- and oximetry-based approaches [19, 20] have demonstrated feasibility in large cohorts; the present work focuses on a stepwise pipeline emphasizing robust ECG signal processing and artifact-tolerant feature extraction as a foundation for future ICU-specific validation.

## Methods

### Study design and dataset

For the development of our analytical tools, we utilized data from The Multi-Ethnic Study of Atherosclerosis (MESA) study, an NHLBI-sponsored 6-center collaborative longitudinal investigation of factors associated with the development of subclinical cardiovascular disease and the progression of subclinical to clinical cardiovascular disease in 6,814 Black, White, Hispanic, and Chinese-American men and women aged 45–84 at baseline in 2000–2002 [22, 23]. A total of 2,237 participants were enrolled in the MESA Sleep Exam (MESA Sleep) that included the collection of full overnight unattended polysomnography (PSG) data that is used in this preliminary study. Data from the MESA study was chosen specifically for its dataset of patients that included balanced diversity of age, race/ethnicity and sex. The MESA sleep data stored in EDF (European Data Format) was downloaded from the National Sleep Research Resource (NSRR) [29]. EDF supports the storage of physiological signals such as ECG, electroencephalogram (EEG), flow, respiration, oxygen saturation (SpO2), electrooculogram (EOG), electromyography (EMG), etc. each at their appropriate sampling rate [24]. The standard EDF does not support clinical annotations and sleep scoring data of the study. This information has been stored in the XML data format in an auxiliary file using clinical review software after the sleep study and during the clinical evaluation.

Of the 2,056 MESA Sleep participants included in the dataset, a sample of 122 participants was selected. The stratified random sampling was used to slightly enrich for patients with AHI > 30 and designed to represent an otherwise normal distribution of OSA severity. For the sample population, descriptive statistics were used to summarize demographic, clinical, and PSG data. Subjects were compared across common AHI severity cutoffs to evaluate between-group differences. Continuous variables were compared using the Kruskal-Wallis rank sum test, while categorical variables were analyzed with Chi-squared or Fisher’s exact test.

### Signal processing

**Fig. 1:**
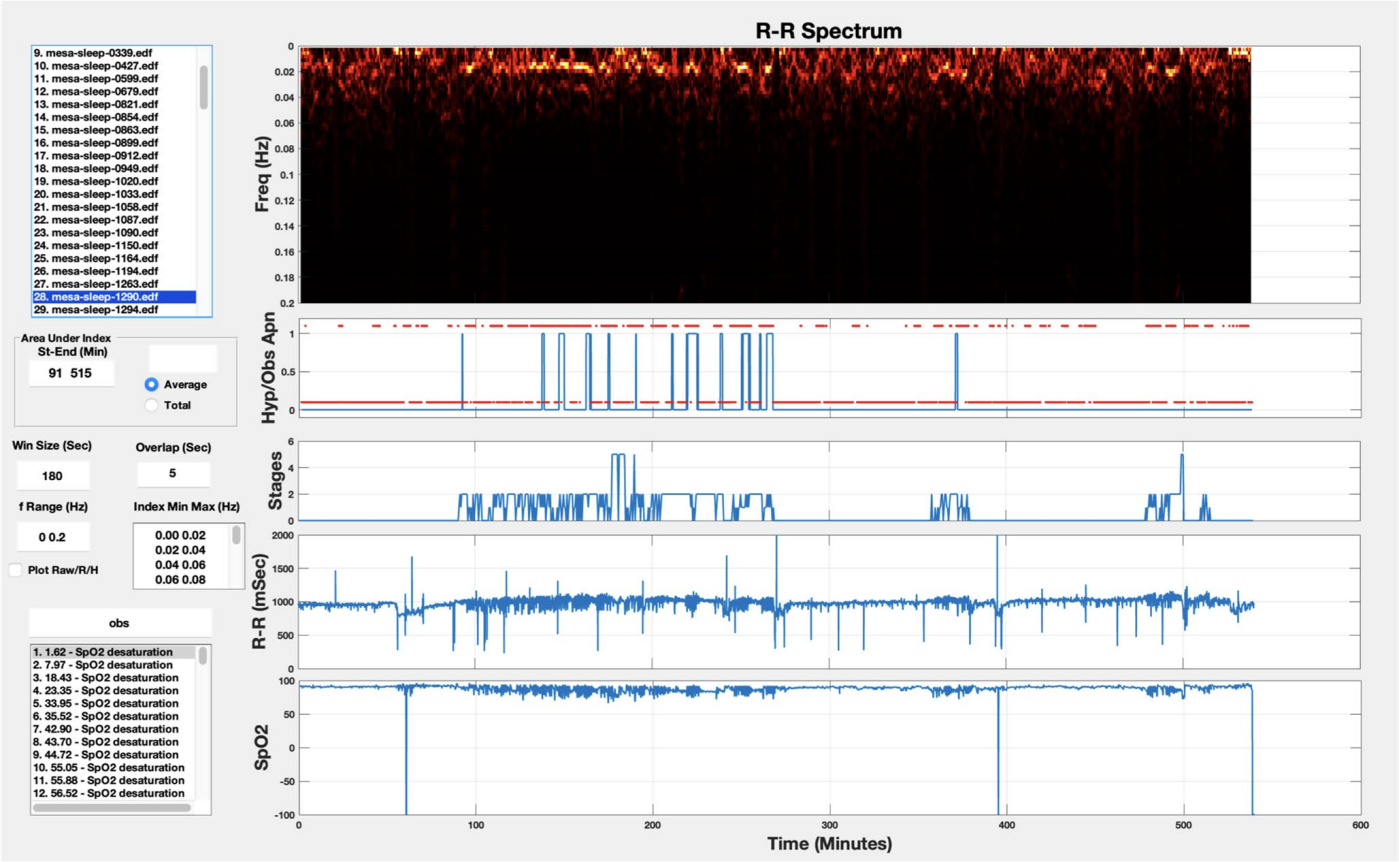
Matlab GUI for loading, visualizing and analyzing the MESA Sleep data

**Fig. 2:**
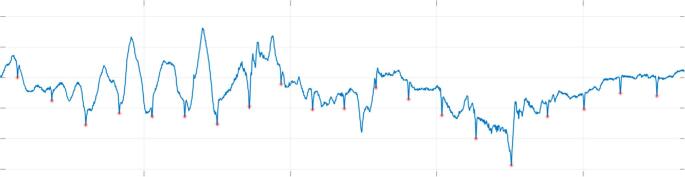
Successful R-wave peak detection within the raw ECG signal with significant motion artifact

Raw ECG data from the EDF data file was used, and respiratory events were identified from the annotations, in particular, the hypopnea and obstructive apnea events. The sampling rate of the ECG data was 256 samples per second and each respiratory event was identified by a start time and duration. To facilitate the visualization, data processing and analysis of the MESA Sleep data, a Matlab GUI was developed (Fig. 1). The first step in analyzing the ECG signal is R-wave peak detection using a robust heartbeat detection algorithm based on time-frequency decomposition [25] that has been validated on more than thousands of hours of clinical patient data [26, 27]. An example of this algorithm extracting R-wave peaks in the midst of motion artifact is shown in Fig. 2.

Once the R-wave peaks were detected, the next step was frequency domain analysis of the R-R (interbeat interval) times-series data. For stationary time-series signals with equally spaced (uniformly sampled) data, the frequency spectra of the signal over the stationary time window is computed using the Fast Fourier Transform (FFT). However, the R-R interval time-series data is non-stationary and is not uniformly sampled (i.e., the time between two consecutive heartbeats is not constant). Thus, a windowing technique and a method for calculating the spectral representation of unevenly sampled data [28] was used for the spectral analysis of the R-R time-series in our approach, and the evolution of the frequency content of the R-R time-series was visualized using the *spectrogram*.

The *spectrogram* is a visual representation of the frequency content of a time-series signal as it evolves over time and provides insight into how the signal’s frequency components are changing over time, allowing for both time- and frequency-domain analyses simultaneously. The computation of the spectrogram involves the following:

1. Windowing-Computing the Fourier transform of the entire signal would not reveal how the frequency content changes over time. Instead, the signal is decomposed into smaller, possibly overlapping time segments, or “windows,” each of which is treated as though it is a stationary signal. Within each window the signal is analyzed separately. Typical windowing functions include Hamming, Hanning, or rectangular window. Overlapping the windows ensures smoother transitions between segments and prevents loss of information at the boundaries of each window. The window size is chosen based on the frequency resolution that is required for the analysis.
2. Because the heartbeat/R-R interval data is not uniformly sampled, the Lomb-Periodogram is used to analyze the frequency content of each “stationary” window.
3. The spectrogram is a 2D plot where the *x-axis* represents time, the *y-axis* represents frequency, and the *color or intensity* indicates the magnitude of a particular frequency at a particular time. The spectrogram essentially displays how spectral content evolves over time.

**Fig. 3:**
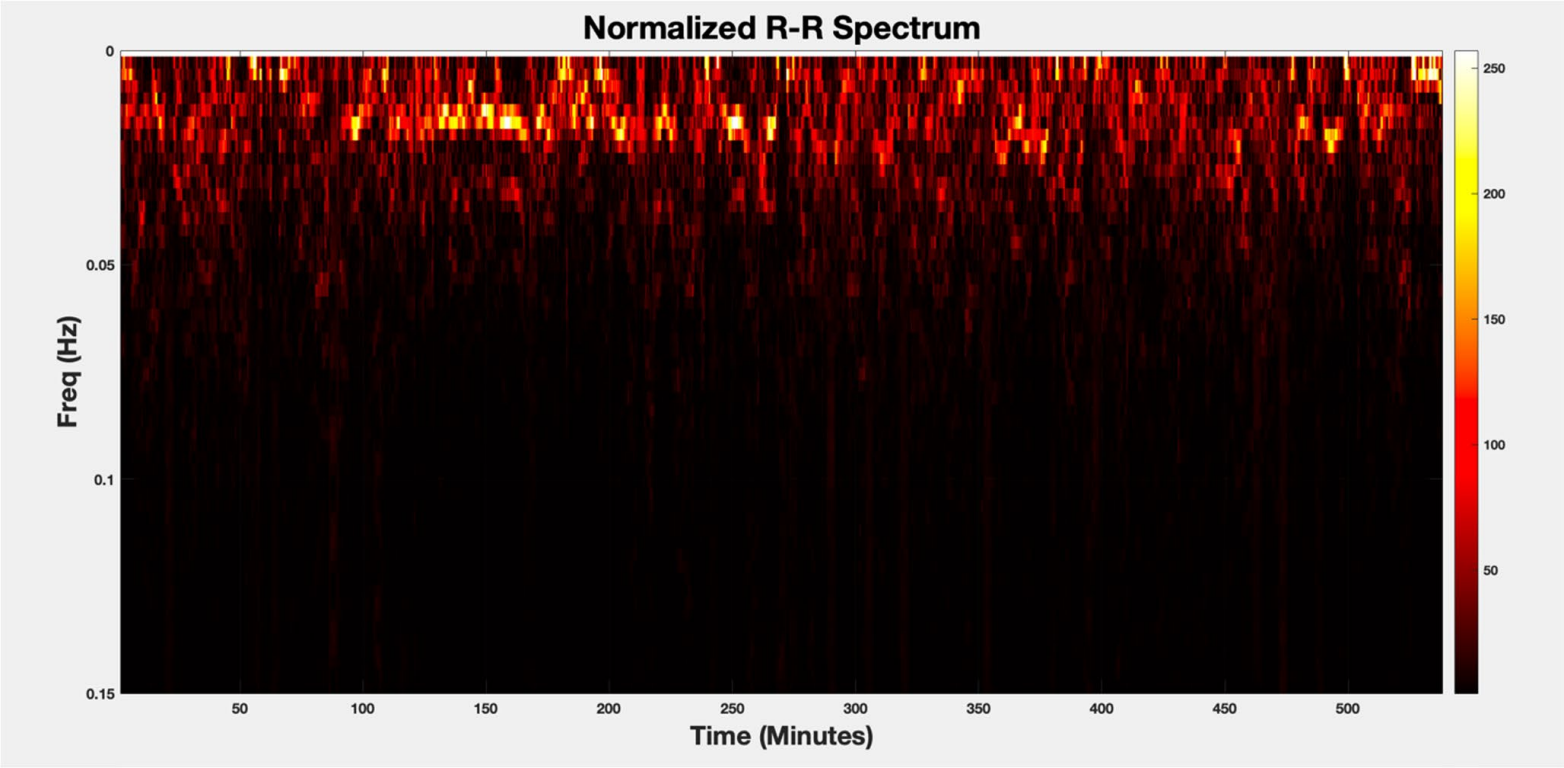
The spectrum of R-R intervals for a sample sleep study (Window Size = 180 s, Overlap 175 s)

An example of the normalized spectrum of the R-R interval time-series for an entire sleep study is shown in Fig. 3. The spectral analysis window size is 180 s with 175 s of overlap for each consecutive pair of windows. The 180-seconds window size provides a frequency resolution of ~ 0.006 Hz.

### Feature extraction and classifier development

For this preliminary study we have chosen to use one of the traditional ML methods for classification, Support Vector Machines (SVMs) a powerful class of supervised learning algorithms primarily used for classification (29). The core idea behind the SVM is to find a separating surface, e.g. a linear hyperplane (or decision boundary) or a non-linear surface that best separates the data points into different classes. In our application, the SVM is a binary classifier, where the time-series data is classified into one of two categories/classes. The algorithm aims to find the best linear hyperplane (linear SVM) of more generally surface (non-linear SVM) that separates the data points into two classes with the highest specificity and sensitivity.

The linear SVM is the simplest SVM model and applies to data that is linearly separable in some feature space, that is a straight line (in 2D) or a hyper-plane (in higher dimensions) cleanly separates the data into two classes. In cases where data is not linearly separable, SVMs can still work by using a kernel function that transforms the original feature space into a higher-dimensional space where the data points may become (linearly separable). Common kernel functions include: linear, polynomial, radial basis, and sigmoidal functions. A radial basis (Gaussian) kernel function has been used in this preliminary study, as the feature data was not linearly separable.

**Fig. 4:**
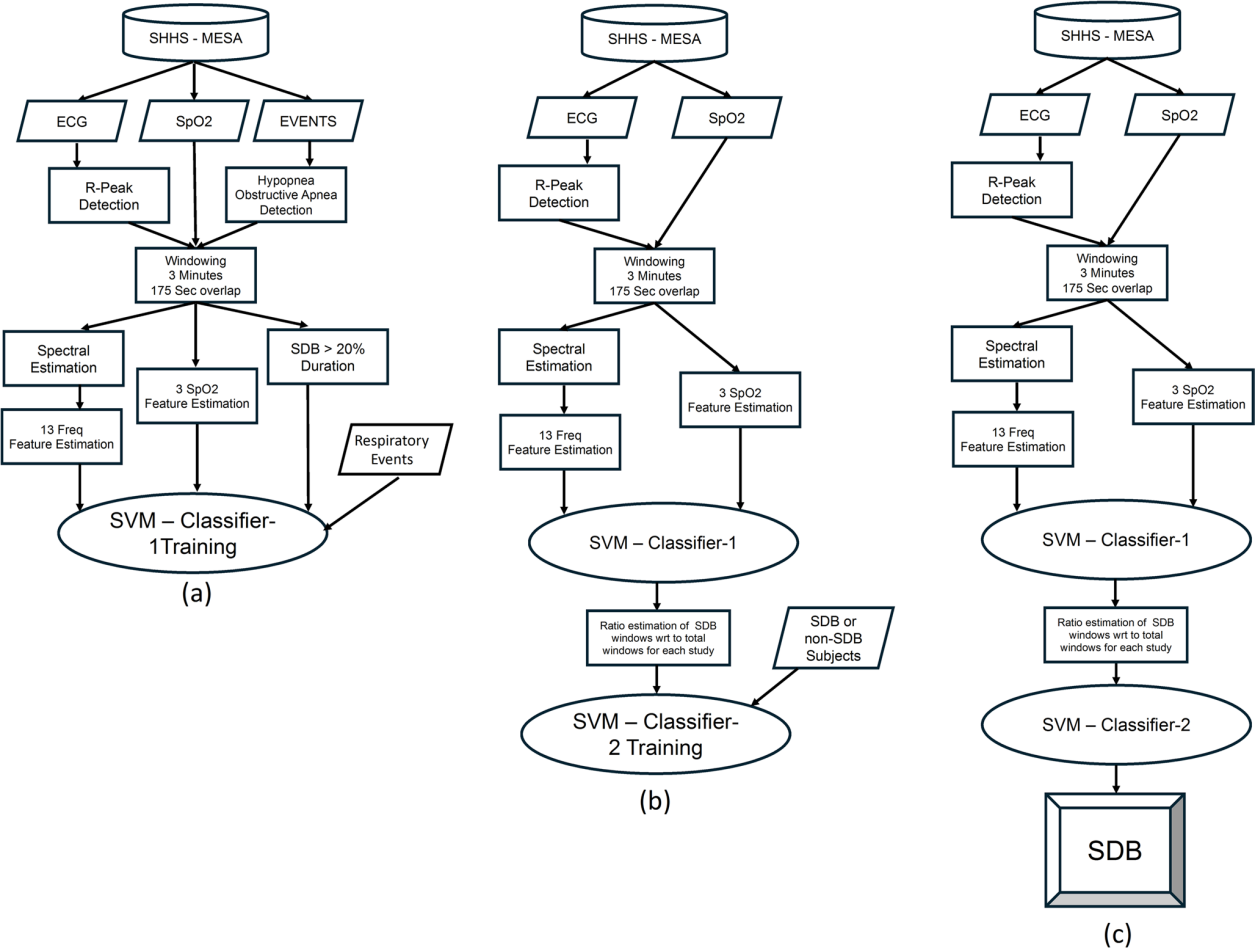
Workflow schema for feature extraction, training and cross validation of the first classifier (SVM-1) in Fig. 7a, feature extraction, training and cross validation of the second classifier (SVM-2) in Fig. 7b, and for testing/validation in the validation set in Fig. 7c

For classification using the preliminary data, the SVM binary classifier uses the Matlab function (fitcsvm). SVM uses a supervised learning paradigm and a set of observations/features as inputs, and the corresponding desired outcomes as outputs for training the classifier. A workflow schema for the steps of developing/validating the classifier is provided in Fig. 4.

The classifier training set included 14 subjects from the sample population that were identified from review of the clinical dataset as true positive (severe OSA) upon clinical evaluations and 15 subjects as true negatives (no OSA). The validation set included the remaining 92 subjects from the sample population not included in the training set.

#### SVM-1

The first SVM classifier assigns respiratory event (hypopnea/apnea) labels (0 if the window did not contain a respiratory event and 1 if the window contains a respiratory event) to each observation (window of ECG and SpO_2_ waveform data). The respiratory event annotations of subjects were carefully analyzed to identify the windows (time segments) that include respiratory events. The window size for analysis for the spectral calculation was set as 180 s to provide the necessary frequency resolution for heart rate variability analysis. This window size is significantly larger than the typical duration of the respiratory events. Therefore, based on prior analyses of respiratory event duration in a large population-based dataset [30], each window with at least 36-seconds (20% of the window length) that included respiratory events was labeled as an SDB window for SVM training and testing. Subsequently, all 180-second windows with 175-seconds overlap were analyzed to identify the set of SDB observations.

In total, 24,455 windows were identified as an SDB (positive) window. As a comparator, 24,679 windows, were randomly chosen from the normal (negative) non-SDB subjects for training and testing the classifier.

The SVM inputs for each window included the average of the normalized magnitude of the frequency bins within 13-frequency bands of the R-R intervals spectrogram as follows: from 0 Hz to 0.02, from 0.02 Hz to 0.04, from 0.04 Hz to 0.06 Hz, from 0.06 Hz to 0.08 Hz, from 0.08 Hz to 0.10 Hz, from 0.10 Hz to 0.12 Hz, from 0.12 Hz to 0.14 Hz, from 0.14 Hz to 0.16 Hz, from 0.16 Hz to 0.18 Hz, from 0.18 Hz to 0.20 Hz, from 0.20 Hz to 0.25 Hz, from 0.25 Hz to 0.30 Hz, and from 0.30 Hz to 0.35 Hz. In addition to these frequency domain features, the following features from the SpO_2_ waveform were also considered: the difference between the maximum and minimum SpO_2_ values, mean SpO_2_, and the standard deviation of SpO_2_ in each window.

For developing the SVM-1 classifier, the Gaussian or radial basis kernel function along with the standardize and kernel auto scale options were used. After training the model, the performance of the classifier was validated using 10-fold cross validation.

#### SVM-2

The purpose of the second SVM classifier was to assign labels to each subject analyzed (0 for non-SDB and 1 for SDB). The same training set of 30 subjects used in SVM-1 were included. The inputs for SVM-2 were chosen as the ratio of the number of SDB windows (identified as having respiratory events detected by SVM-1) to the total number of windows that were analyzed (analogous to % time in apnea/hypopnea), and the outputs were chosen as the presence/absence of an OSA diagnosis for the subjects as determined by the previous clinical evaluation.

After training the model, the performance of the classifier was validated using 10-fold cross validation.

#### Validation set

Next, we evaluated the ability of the model to classify subjects outside of the training set. The records of the remaining 92 subjects from the sample population were provided as inputs to the SVM classifiers with binary classification outputs defined as 0 (non-SDB) or 1 (SDB). Given the median AHI of the sample population was just less than 20 events/h, the remaining subjects were classified according to their AHI (from the clinical dataset) as positive (AHI > 20) or negative (AHI < 20) for SDB for the purposes of performance evaluation.

### Performance evaluation

To evaluate the performance of our models, confusion matrices of each iterative step were created and used to calculate sensitivity, specificity, positive predictive value (PPV), negative predictive value (NPV), and F1 score. For sensitivity, specificity, PPV, NPV, and overall accuracy, 95% confidence intervals (CIs) were calculated using Wilson score intervals for binomial proportions.

## Results

### Sample population

Records from 122 participants were analyzed. The median age of the sample was 69 years (IQR, 62–75) and 63 (52%) were male. The sample had representation across the four identified race/ethnicity groups: Caucasian (35%), Black, African-American (9%), Chinese-American (24%) and Hispanic (32%). The median BMI was 28.2 (IQR, 24.7–31.9). The mean AHI was 26.5 ± 22.0 and 17.3 ± 19.6 using the AASM and CMS criteria, respectively. There were no significant differences in any of the demographic or sleep data between the training set compared to the validation set (all comparisons *p* > 0.05). Further details on demographic, clinical and PSG data are included in Tables 1 and 2.

**Table 1:**
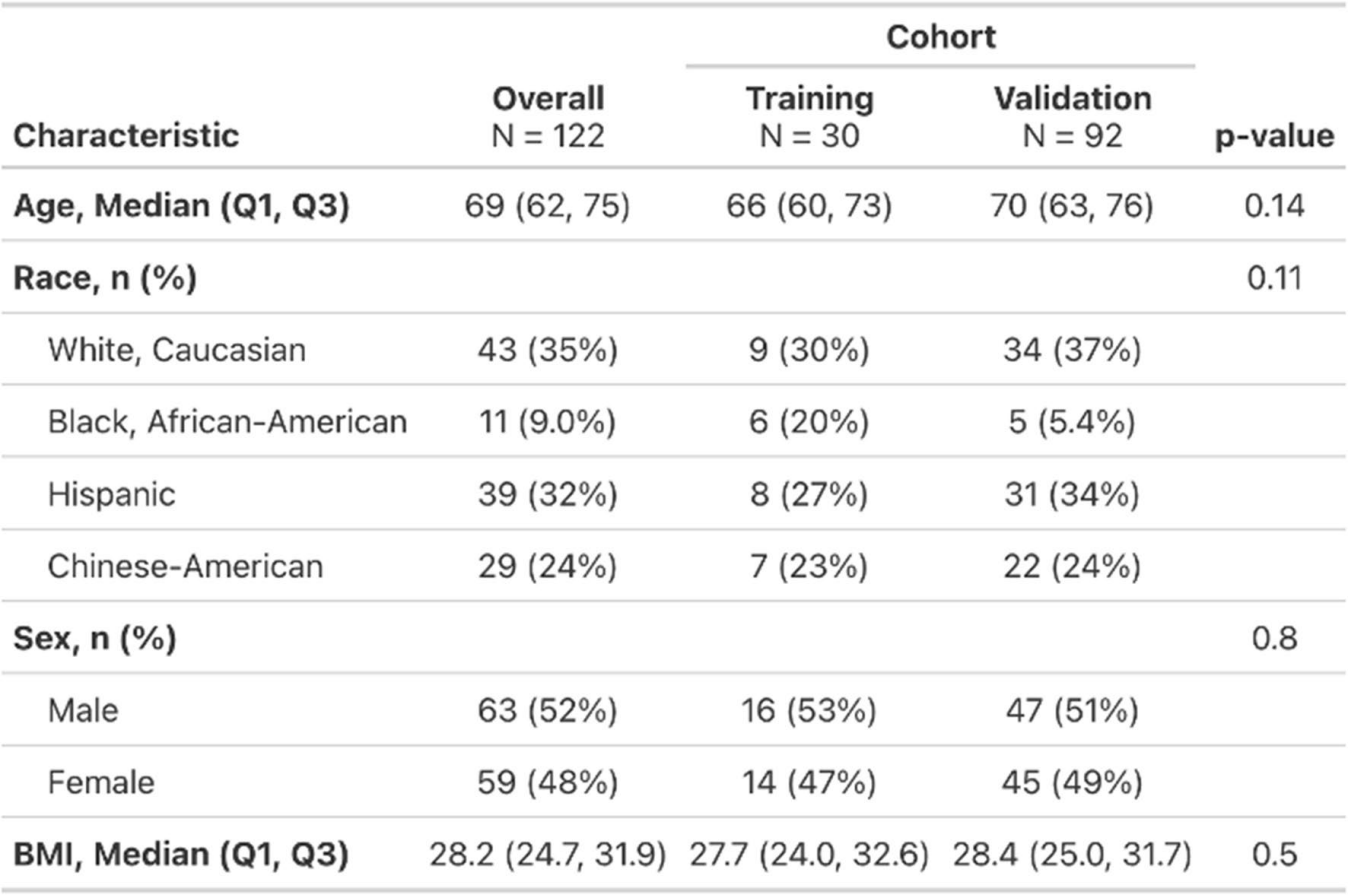
Participant demographics by cohort (training vs. validation). Values are reported as median (Q1, Q3) for continuous variables and n (%) for categorical variables. P-values compare training and validation cohorts using the Wilcoxon rank-sum test for continuous variables and pearson’s chi-square test for categorical variables

**Table 2:**
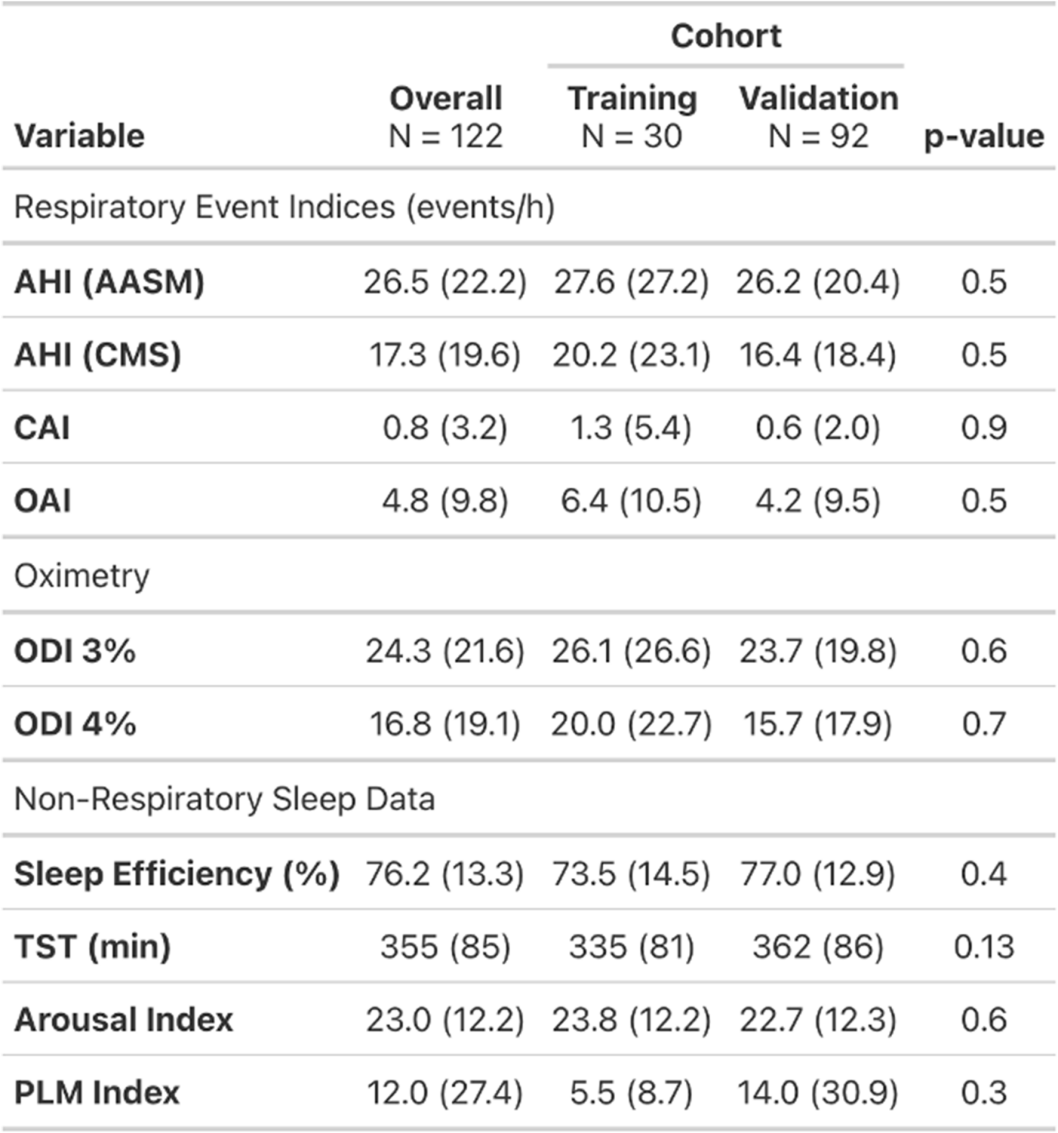
Polysomnography-derived sleep and respiratory characteristics by cohort (training vs. validation). Continuous variables are reported as mean (SD). P-values compare training and validation cohorts using the Wilcoxon rank-sum test

## Classifier performance

### Training

#### SVM-1

**Fig. 5:**
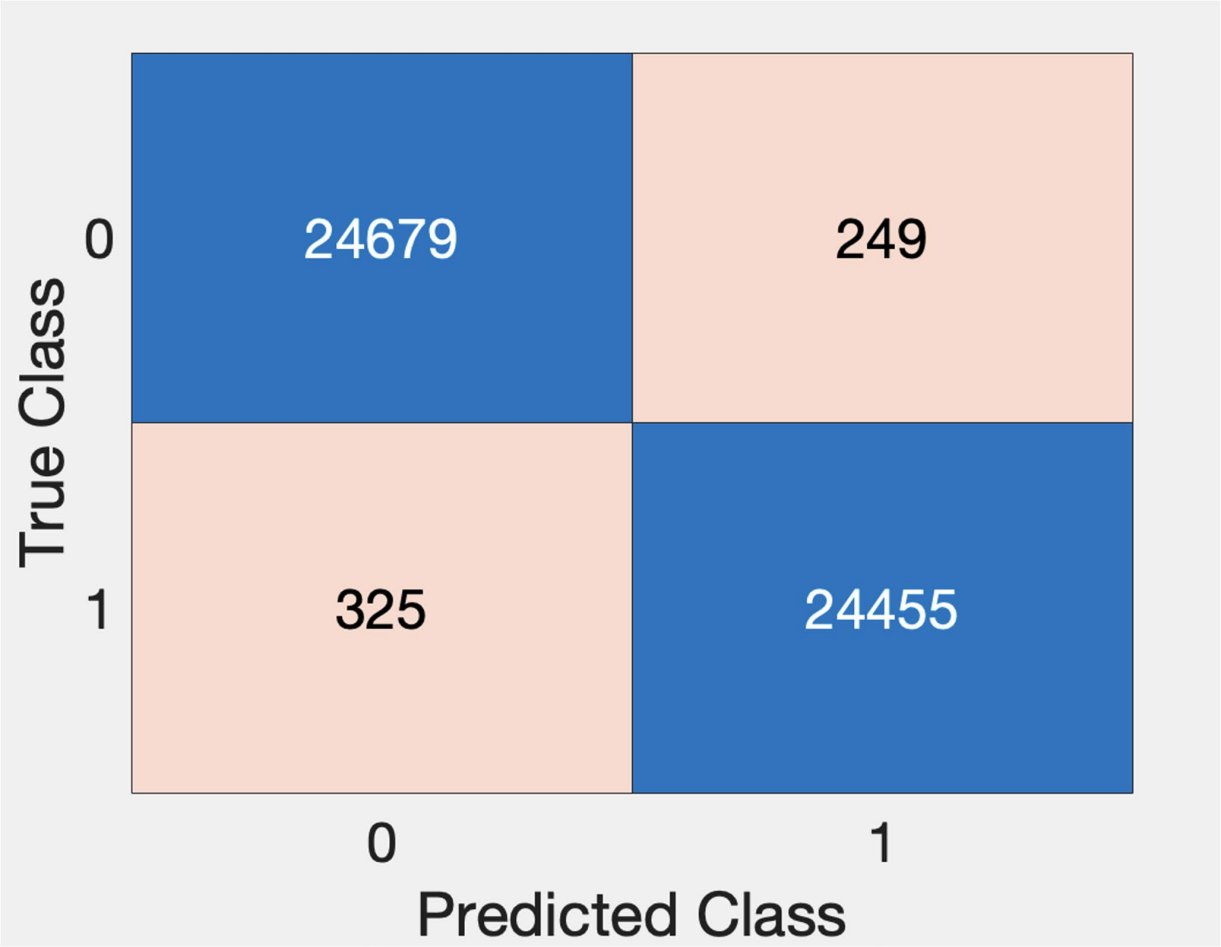
Confusion Chart for SVM-1 for window-by-window analysis of the training dataset as non-SDB (0) or SDB (1)

The SVM-1 classifier had a sensitivity of 98.7%, specificity of 99.0%, PPV of 99.0%, NPV of 98.7% and F1 score of 98.8% in its ability to label windows as having the presence or absence of SDB events (Fig. 5).

#### SVM-2

**Fig. 6:**
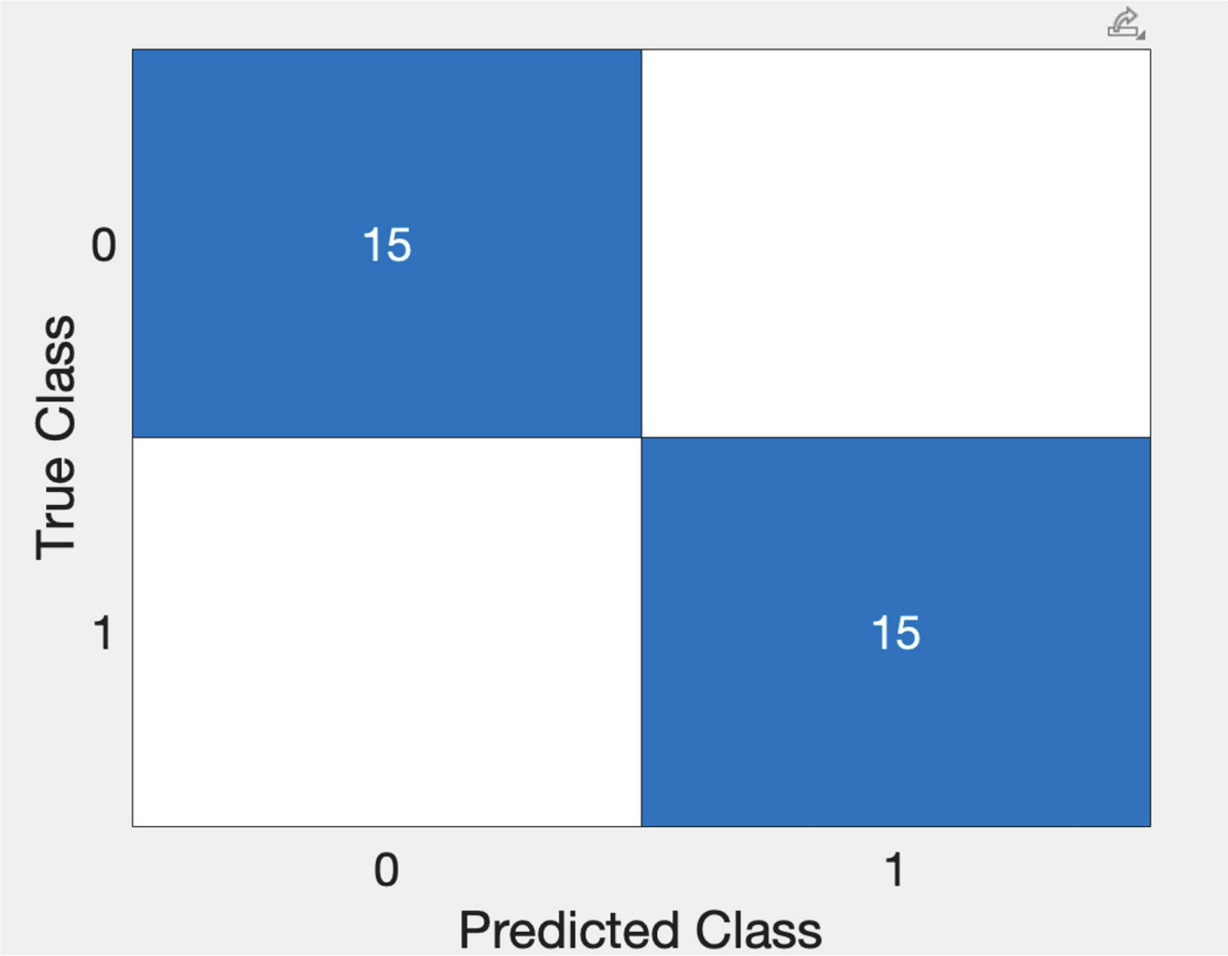
Confusion Chart for SVM-2 for classification of subjects as non-SDB (0) and SDB (1)

The SVM-2 classifier had a sensitivity of 100.0%, specificity of 100.0%, PPV of 100.0%, NPV of 100.0% and F1 score 100.0% for classifying the training set subjects’ PSG as showing presence of severe OSA or no significant sleep-disordered breathing (Fig. 6).

#### Validation

**Fig. 7:**
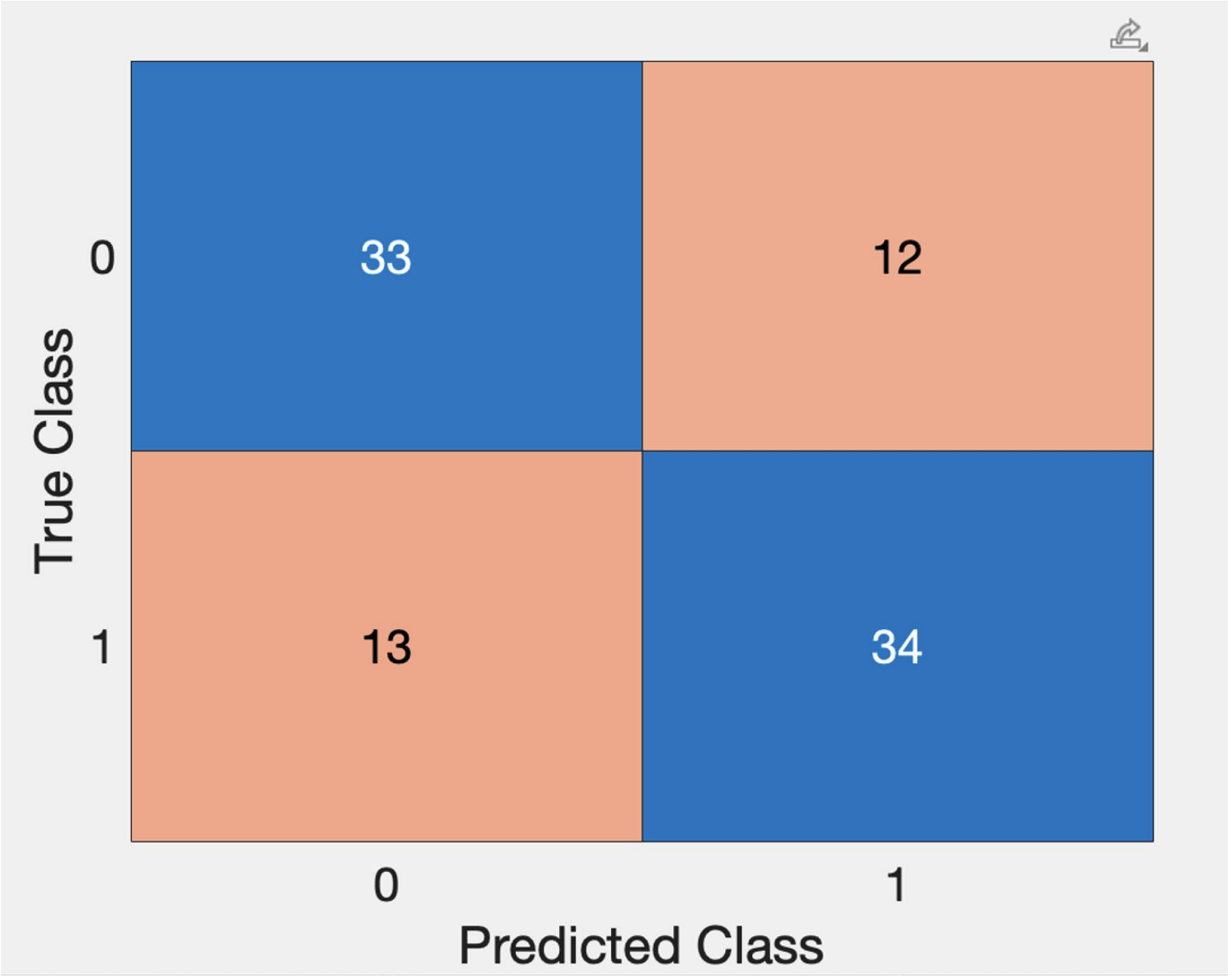
Confusion Chart for Validation Set using SVM-1 and SVM-2, stepwise with subjects classified as SDB negative (0) and SDB positive (1)

On subject-level classification in the validation cohort (*n* = 92), the combined model (Fig. 7) demonstrated a sensitivity of 72.3% (95% CI, 57.4%–84.4%) and specificity of 73.3% (58.1%–85.4%). PPV was 73.9% (58.9%–85.7%) and NPV was 71.7% (56.5%–84.0%). Overall accuracy was 72.8% (62.6%–81.6%), with an F1-score of 73.1%.

## Discussion

We developed a machine-learning classifier capable of differentiating subjects with and without significant sleep-disordered breathing (SDB) in a curated dataset using only electrocardiogram (ECG) and pulse oximetry (SpO₂) waveforms. The purpose of the study was to implement and test a classification tool that relied only on physiological signals routinely measured in the MICU setting that could be used in patient management. The results show significant promise but identify some potential limitations in validation related to spontaneous arrhythmias and incomplete capture of respiratory effort.

By leveraging typically collected physiological signals, our approach offers valuable information regarding breathing over time using ECG and oximetery, common signals monitored in hospitalized patients, particularly those in the intensive care unit (ICU). The high sensitivity and specificity observed in the training set underscore the potential of this methodology in detecting SDB-related physiological signatures. Although this is not completely novel and has been attempted in some HST approaches, the goal here is to effectively implement this in an intensive care unit without additional hardware. The modest performance in the validation set suggests the need for further refinements, particularly in handling dimensions of inter-individual variability in autonomic and cardiovascular responses to respiratory disturbances. Our findings reinforce the feasibility of non-intrusive, real-time monitoring tools that could be integrated into hospital-based clinical decision support (CDS) systems to facilitate early identification and intervention.

Time-series classification in a machine learning (ML) framework involves predicting a label or category for a sequence of data points collected over time. There are a variety of ML methods that have been developed and applied to time-series classification, ranging from traditional algorithms to more recent deep learning models, and include: traditional ML methods, distance-based methods, deep learning (DL) methods, ensemble methods, and hybrid methods [31, 32].

Examples of traditional ML methods include: Support Vector Machines (SVMs), Random Forest (RF), K-Nearest Neighbors (KNN); examples of distance-based methods include: dynamic time warping (DTW) and K-means clustering; examples of DL methods include: Recurrent Neural Networks, Long-Short Term Memory Networks (LSTM), Gated Recurrent Units (GRUs), Convolutional Neural Networks (CNNs), and Transformer Models. Examples of ensemble methods include: Gradient-Boosting Machines (GBMs including XGBoost and LightGBM. Hybrid approaches are methods that combine multiple models or techniques to leverage the strengths of each, for example: DL combined with feature extraction where DL is used for feature extraction and traditional ML methods are used for classification, and ensemble time-series models where multiple time-series models are combined to improve the robustness and accuracy of the classification [33].

The choice of machine learning method for time-series classification depends on several factors such as the amount and type of data (e.g., univariate or multivariate, frequency of observations), computational resources, and the importance of interpretability. For simple, smaller datasets, traditional methods like SVM or Random Forest are good choices. For more complex and larger datasets with significant temporal dependencies, deep learning methods like LSTMs, GRUs, or Transformers may be more effective. Hybrid and ensemble approaches can often provide improved performance by combining the strengths of different ML models [34].

### Strengths

A key strength of this study is its use of a curated dataset with full polysomnography (PSG) validation, allowing robust evaluation of ECG/SpO2-based signal processing techniques. The classifier was trained using a randomized sample that reflects real-world variations in demographics and OSA severity, enhancing its generalizability. Additionally, by employing spectral analysis of heart rate variability and SpO₂ fluctuations, we capitalized on established physiological markers of SDB. Our stepwise machine-learning approach that first classifies respiratory events in time-series windows and subsequently aggregates data to assign patient-level labels, aligns with established understanding of OSA pathophysiology and identification. This hierarchical methodology is adaptable and could be refined further to improve classification accuracy.

### Limitations

Despite these strengths, several limitations should be acknowledged. First, ECG-based SDB detection is inherently limited in cases of cardiac arrhythmias, particularly frequent premature atrial or ventricular contractions (PACs/PVCs) that can distort heart rate variability-based spectral analyses. Although atrial fibrillation was less disruptive, ectopic beats frequently led to uninterpretable spectrograms, reducing model reliability in patients with underlying dysrhythmias. Detecting ECG artifacts, physiological or motion induced, is critical. The algorithm implemented in this study for R-beat detection uses an innovative signal processing approach based on the complex wavelet transform. The algorithm includes artifact defection, and has been validated in numerous clinical studies [26, 27, 35]. Second, while SpO₂ provides valuable adjunctive data, the absence of direct respiratory effort measures (e.g., thoracic impedance, nasal airflow) may lead to misclassification in milder cases of SDB. Additionally, an important limitation of this study is that it was developed and tested using biosignals from unattended PSGs with robust methodology and signal quality. ICU data is likely to include greater artifact and instability/variability inherent to critical illness that may impact direct translation. Future clinical trials using ICU monitoring systems may require additional signal pre-processing or model adjustments to achieve similar performance. Lastly, the relatively small validation cohort and its enrichment for severe OSA may have biased performance metrics, highlighting the need for external validation in larger datasets.

#### Future directions/ICU translation

While our approach leverages signals that are routinely available in hospitalized and ICU patients, the present study should be viewed as a feasibility pilot developed in a high-quality PSG dataset. Future efforts should focus on collection ICU-acquired ECG/SpO₂ data to quantify performance under real-world artifact, refine preprocessing, and perform external validation prior to clinical decision support deployment. Subsequent iterations should explore multimodal feature extraction, integration of additional biosignals, and possible expansion of machine learning methodology to refine classification accuracy and clinical applicability. Additionally, best practices for ICU translation and implementation into clinical decision support systems should be thoroughly investigated to maximize uptake and impact of these novel tools in order to make meaningful change to patient care.

## Data Availability

Data available on request from the authors. The data that support the findings of this study are available from the corresponding author, ZS, upon reasonable request.
